# Anesthetic approaches for malpresentation and multiple gestation

**DOI:** 10.1097/ACO.0000000000001614

**Published:** 2026-02-12

**Authors:** Oscar F.C. van den Bosch, Feline F.J.A. ter Bruggen, Erica M. Johnson, Elizabeth M.S. Lange

**Affiliations:** a Wilhelmina Children’s Hospital, University Medical Center Utrecht, Utrecht, The Netherlands; b Grady Memorial Hospital; c Emory University Hospital Midtown, Emory University School of Medicine, Atlanta, Georgia, USA

**Keywords:** breech, malpresentation, multiple gestation, obstetric anesthesia

## Abstract

**Purpose of review:**

This review provides an updated overview of anesthetic considerations for malpresentation and multiple gestation. Key topics include analgesia for external cephalic version (ECV), labor analgesia for vaginal delivery in malpresentation and multiple gestation, anesthetic considerations for cesarean delivery in these scenarios, and management of postpartum hemorrhage risk.

**Recent findings:**

Neuraxial analgesia improves both maternal comfort and procedural success during ECV for malpresentation. Neuraxial labor analgesia facilitates safer vaginal delivery in multiple gestation pregnancies. Cesarean delivery for multiple gestation carries an increased risk of uterine atony, with higher oxytocin requirements (ED_90_ > 4 IU) compared with singleton pregnancies.

**Summary:**

Anesthesiologists should maintain vigilance and readiness for rapid intervention when caring for patients with malpresentation and/or multiple gestation. Persistent gaps exist between recent evidence and routine clinical practice; therefore, implementation studies and multidisciplinary consensus guidelines are warranted.

**KEY POINTS**

- Malpresentation and multiple gestation require readiness for rapid anesthetic intervention.
- Neuraxial analgesia improves comfort and success of external cephalic version, yet most external cephalic versions are performed without analgesia.
- Epidural analgesia supports safe vaginal twin delivery.
- Cesarean twin deliveries carry a higher uterine atony risk and require higher oxytocin doses (ED95 > 4 IU) than singleton pregnancies.
- Research-practice gaps persist; therefore, implementation trials and consensus guidelines are warranted.

Malpresentation and multiple gestation are common obstetric scenarios that present distinctive challenges for the anesthesiologist. They both carry higher rates of perinatal morbidity and mortality. When complications arise, obstetric emergencies may necessitate rapid anesthetic intervention. This requires clear communication and coordinated decision-making among the obstetrician, anesthesiologist, and patient. This focused review examines recent findings in obstetric anesthesia with particular emphasis on evidence published in the past 5 years.

## MALPRESENTATION

### Breech mode of delivery

Breech presentation refers to a fetal lie in which the buttocks, feet, or both present at the maternal pelvic inlet rather than the head. Most malpresentations rotate spontaneously to a cephalic presentation before 34 weeks of gestation. At term, however, 3–4% of singleton pregnancies will remain in a breech position. The optimal mode of delivery continues to be debated, and significant geographical differences persist, even among high-income countries.

The Term Breech Trial randomized 2083 patients with a term breech fetus to either a planned cesarean delivery or planned vaginal delivery. The trial demonstrated a lower rate of perinatal or neonatal mortality and serious neonatal morbidity in the planned cesarean delivery group [1.6 vs. 5.0, relative risk: 0.33, 95% confidence interval (CI): 0.19–0.56], which contributed to a global shift toward planned cesarean delivery in the setting of breech presentation [1–3]. However, at 2-year follow-up, no significant differences were observed in maternal or pediatric outcomes [4,5]. Most recent data remain mixed. A study from a dedicated breech birth team reported that 78.9% of breech presentations were managed with planned cesarean delivery. Among those who attempted vaginal delivery, 21% ultimately required intrapartum cesarean delivery [6]. Despite careful selection and experienced management, planned vaginal delivery was still linked to a higher risk of short-term neonatal mortality and morbidity compared with cesarean delivery (3.1 vs 0.7%) [6]. In contrast, a large registry-based cohort study from Norway found no increased risk of perinatal death or cerebral palsy with vaginal breech delivery [7]. National guidelines on the contraindications for vaginal breech delivery vary widely [8^■^]. In preterm and extremely preterm breech deliveries, planned cesarean delivery is linked with lower neonatal mortality, as shown in recent population-based cohort studies and confirmed in a systematic review and meta-analysis [9^■^,10^■^]. Patient counseling regarding mode of delivery should include shared decision-making and consider team expertise, institutional resources, and coexisting risk factors for intrapartum fetal compromise.

### External cephalic version

The goal of external cephalic version (ECV) is the manual rotation of the fetus from the breech or transverse position to a cephalic presentation, thereby reducing the likelihood of cesarean delivery and its maternal risks. During the procedure, uterine relaxation may be achieved with β2 adrenergic receptor agonists such as terbutaline or with the oxytocin receptor antagonist atosiban. It is typically performed after 36–37 weeks of gestation, and the maternal discomfort can be substantial, with reported pain scores averaging 5.6 (interquartile range 2.7–6.8) on a 0–10 scale [11]. Potential complications include fetal bradycardia, umbilical cord prolapse, maternal hemorrhage, and placental abruption [12]. Analgesic techniques, including neuraxial, intravenous, or inhalational approaches such as the use of nitrous oxide, may improve maternal comfort and procedural success. The use of neuraxial analgesia specifically increases preparedness for fetal emergencies associated with ECV, such as placental abruption, hemorrhage, or cord prolapse. The advantages, considerations, and side effects of neuraxial analgesia for ECV are shown in Table 1.

**Table 1.** Summary of advantages, considerations, and side effects of neuraxial analgesia for external cephalic version

Advantages	Considerations/side effects
Improves maternal comfort	Requires additional personnel and resources
Reduces force required for version	May prolong recovery time
Increases version success rate	Potential for transient maternal hypotension
May increase likelihood of vaginal delivery	Risk of post-dural puncture headache (1 : 100)
Improves readiness for emergency delivery^a^	

a When the neuraxial block is of adequate spread and depth, or a catheter is in place.

Although most ECVs are performed without analgesia [13], a recent survey reported that patients who received neuraxial analgesia would recommend it to others, and that greater analgesic efficacy correlated with increased comfort, potentially encouraging wider adoption of ECV as an alternative to planned cesarean delivery [14^■^].

Multiple trials have evaluated the effects of analgesia on ECV success. Three meta-analyses assessing 19, 17, and 9 studies, respectively, confirmed higher success rates with analgesia, both neuraxial and intravenous, compared with no analgesia [15^■■^–17]. An observational study suggests that less force is required to achieve successful ECV under neuraxial analgesia [18]. The optimal neuraxial approach remains debated [19]: intrathecal techniques provide a dense and reliable block, whereas epidural catheters allow rapid extension to surgical anesthesia if emergent cesarean delivery becomes necessary.

Reported intrathecal doses range from low-dose bupivacaine (2.5 mg) to anesthetic doses up to 10 mg [16,20]. A randomized controlled trial assigned 240 subjects to 2.5, 5.0, 7.5, or 10 mg of intrathecal bupivacaine, combined with 15 mcg of intrathecal fentanyl. Doses greater than 2.5 mg did not lead to an additional increase in procedural success rate or a lower incidence of cesarean delivery [20]. Analgesic doses appear to be equally effective as anesthetic doses in terms of maternal comfort and uterine relaxation.

Intravenous analgesia with meperidine, fentanyl, or remifentanil (continuous infusion or patient-controlled analgesia) improves ECV success to a lesser degree than neuraxial analgesia [15^■■^,^16^]. Inhalational agents, such as nitrous oxide, have not shown benefit in improving ECV success rates [21,22].

Approximately 79.3% of successful versions result in vaginal birth [23]. As analgesia improves the success rate of ECV, it may also increase the likelihood of subsequent vaginal delivery. Two out of three available meta-analyses found that neuraxial analgesia or anesthesia for ECV increased the likelihood of vaginal delivery, whereas intravenous and inhalational strategies did not [15^■■^–17]. Adequately powered trials are warranted to identify which patients benefit most from analgesia and to identify the most effective route and dosing strategy. For the anesthesiologist, providing neuraxial analgesia for ECV is an opportunity to improve outcomes for both the pregnant patient and the neonate by increasing the likelihood of vaginal delivery.

### Planned vaginal breech delivery

The choice of labor analgesia for vaginal breech delivery should be individualized, considering patient preferences and obstetric risk factors. Benefits of neuraxial analgesia include effective pain relief, inhibition of early expulsive efforts, and facilitation of rapid conversion to anesthesia should instrumental or operative delivery become necessary. In the second stage of labor, effective maternal expulsive efforts are important for vaginal breech birth, and excessively dense epidural blockade from higher concentrations of local anesthetic may impair this balance.

Two recent studies have examined the impact of epidural analgesia on intended vaginal breech deliveries at term. In a 10-year retrospective cohort study from Germany, continuous epidural analgesia was associated with a higher rate of manual assistance during vaginal breech delivery (52.1 vs. 33.8%) and longer labor duration (mean difference 293 min) [24]. However, parity was not controlled for despite a higher proportion of nulliparous patients in the epidural group, which may significantly confound the association between epidural analgesia and delivery outcomes. Cesarean delivery rates were higher with epidural use overall, but this difference disappeared when only primiparous patients were analyzed. A retrospective case-control study from Finland of preterm vaginal breech deliveries found epidural analgesia to be associated with more short-term neonatal morbidity, likely due to differences between patients that requested analgesia and those that did not, for example, its use in longer or more complicated labors [25]. Importantly, no high-quality evidence demonstrates different breech labor outcomes with epidural analgesia, while an indwelling catheter provides the advantage of rapid intervention in the event of a cesarean delivery.

In the USA, vaginal breech deliveries are usually performed in the operating room to ensure immediate access to cesarean delivery if needed. Vaginal breech deliveries may be conducted in the delivery room to enhance maternal comfort, provided that an anesthetic team and an operating room are readily available and within proximity. Neuraxial labor analgesia in breech delivery presents a particular challenge: analgesia must provide sacral coverage while preserving maternal expulsive efforts. The optimal neuraxial initiation technique remains under debate. Epidural maintenance requires frequent assessment, ideally every 2–4 h, to ensure that the block can be rapidly converted to anesthesia if cesarean delivery is needed [26^■^,27^■■^].

On occasion, instrumental vaginal delivery for the breech infant may be necessary with the use of Piper forceps, which requires rapid extension of neuraxial analgesia. The use of 3% 2-chloroprocaine or 2% lidocaine with epinephrine and bicarbonate is a suitable choice for the conversion of epidural analgesia to anesthesia. These concentrated solutions should be administered at the first sign of difficulty with the delivery of the fetus.

The most concerning obstetric complication in vaginal breech delivery is head entrapment. Uterine relaxation may be requested and can be rapidly achieved with nitroglycerin, administered sublingually (800–1600 mcg) or intravenously (100–300 mcg). Alternatively, the oxytocin receptor antagonist atosiban is frequently used in some countries to achieve uterine relaxation without the hemodynamic side effects associated with nitroglycerin use, though it is not available in the USA. Alternatives include terbutaline, a β2 adrenergic receptor that assists in tocolysis. Only rarely, is general anesthesia with volatile anesthetics necessary to provide sufficient relaxation for extreme cases of fetal head entrapment. In addition to uterine relaxation, head entrapment may require rapid extension to surgical anesthesia to allow instrumental or operative interventions. In the absence of an indwelling epidural catheter, general anesthesia is typically the only viable option. Anesthesiologists should therefore ensure the availability of appropriate drugs and airway equipment whenever a breech vaginal delivery is planned.

### Planned cesarean delivery for breech presentation

Fetal malpresentation remains a common indication for planned cesarean delivery, accounting for 18.5% of primary cesarean deliveries [28]. Anesthetic management is similar to that for cephalic presentations and most often involves single-shot spinal or combined spinal-epidural anesthesia, in the absence of contraindications to neuraxial anesthesia. The obstetrician may request additional uterine relaxation, for which nitroglycerin should be readily available. Only in extreme cases may general anesthesia be necessary for uterine relaxation, which is best achieved with high concentrations of volatile anesthetics. When uterine relaxants are used, clinicians should anticipate an increased risk of uterine atony and postpartum hemorrhage (PPH) and be prepared to administer additional uterotonics as needed.

## MULTIPLE GESTATION

Twin pregnancies occur spontaneously in ~1 in 250 pregnancies, triplet pregnancies in about 1 in 10 000, and quadruplet pregnancies in 1 in 700 000 pregnancies. Multiple gestation is associated with increased morbidity, including preterm birth, uterine overdistention and atony, PPH, vascular and placental complications such as placental abruption or cord prolapse, and a higher likelihood of emergency cesarean delivery for suspected intrapartum asphyxia, particularly for the second neonate. Current evidence supports a trial of labor when the presenting twin is in a cephalic position [29^■^]. A randomized controlled trial found no improvement in fetal or neonatal outcomes with planned cesarean delivery compared with planned vaginal delivery [30,31]. However, planned cesarean delivery is generally recommended for monochorionic-monoamniotic twins and when the presenting twin is breech [29^■^]. Overall, multiple gestation is associated with a higher rate of cesarean delivery.

### Vaginal delivery

Neuraxial analgesia provides effective pain relief and, importantly, allows for rapid extension to surgical anesthesia if instrumental or operative delivery of either twin is necessary. For these reasons, most guidelines recommend neuraxial analgesia for the vaginal delivery of multiple gestation [29^■^], and as such, neuraxial analgesia rates are higher in twin than in singleton pregnancies [32]. A recent population-based cohort study from Finland evaluated 3242 twin pregnancies in which the first twin was delivered vaginally [33^■■^]. Epidural analgesia was associated with a reduced rate of cesarean delivery for the second twin [adjusted odds ratio (aOR): 0.64, 95% CI: 0.44–0.92] and a lower rate of neonatal mortality (aOR: 0.52, 95% CI: 0.33–0.79), likely by enabling more robust provider intrauterine manipulation of the second twin to facilitate vaginal delivery. These benefits were accompanied by an increased likelihood of instrumental vaginal delivery (aOR: 1.45, 95% CI: 1.29–1.62). Dural puncture techniques with or without intrathecal drug administration may be beneficial to allow better catheter function and more rapid extension to surgical anesthesia compared with traditional epidural techniques [34]. The choice of neuraxial analgesia initiation technique should consider the patient’s goals, the clinical context, and the institutional culture. For patients who decline or have a contraindication to neuraxial analgesia, intravenous options such as patient controlled analgesia with short-acting opioids may be considered.

Vaginal delivery of twins should be conducted in the operating room or in a delivery room in proximity to an operating room, with an anesthesia provider available throughout. This setup ensures that rapid access to surgical anesthesia should emergency cesarean delivery be required. The availability of neonatal resuscitation teams for each infant is also essential. Regardless of the location of delivery, preparedness for urgent intervention, including adequate intravenous access, established epidural catheter, and immediate access to blood products remains critical to ensuring optimal maternal and neonatal outcomes.

Given the increased risk of intrapartum cesarean delivery and PPH, a well functioning epidural catheter is of critical importance in multiple gestation. Motor and sensory block, pain, and overall clinical status should be assessed at regular intervals (every 2–4 h) [26^■^,27^■■^]. There should be a low threshold to replace poorly functioning catheters, and a dural puncture technique with or without intrathecal drug administration should be considered to enhance onset and efficacy of the block [27^■■^,^34^].

### Cesarean delivery

Neuraxial anesthesia is the preferred anesthetic technique for cesarean delivery in both singleton and multiple gestation pregnancies, and is favored over general anesthesia in the absence of contraindications. Intrapartum cesarean delivery is typically managed with epidural extension of labor analgesia, single-shot spinal anesthesia, combined spinal-epidural anesthesia, or, in rare cases, with general anesthesia, for example, in the setting of emergency or failed neuraxial block.

Historically, multiple gestation was thought to increase cephalad spread of intrathecal local anesthetics, attributed to enhanced sensitivity from elevated progesterone concentrations, increased uterine size, and greater spinal canal compression [35]. More recent evidence challenges this assumption. Two comparative dose–response studies with hyperbaric ropivacaine demonstrated no clinically meaningful difference in spinal anesthetic requirements between singleton and twin pregnancies. At term, the estimated ropivacaine ED_50_ and ED_90_ were 11.2 (95% CI: 10.2–12.0) and 15.7 (95% CI: 14.4–18.3) mg in singletons, and 10.5 (95% CI: 9.5–11.3) and 14.8 (95% CI: 13.6–17.0) mg in twins [36^■■^]. A subsequent trial in preterm pregnancies (<37 weeks’ gestation) confirmed these findings, reporting ED_50_ and ED_90_ values of 9.9 (95% CI: 7.2–11.5) and 16.8 (95% CI: 14.5–22.9) mg for singletons, and 9.2 (95% CI: 6.4–10.8) and 15.6 mg (95% CI: 13.6–20.6) for twins. Hemodynamic parameters, aortocaval compression, and adverse effects were comparable between groups [37^■^].

Multiple gestation is an independent risk factor for postspinal hypotension, which is effectively mitigated with prophylactic vasopressor infusion. Both phenylephrine and norepinephrine provide comparable hemodynamic stability and fetal safety in twin pregnancies, with no clinically meaningful differences in maternal or neonatal outcomes [38^■^,39^■^]. Earlier work showed no difference in vasopressor requirement or hemodynamic instability during spinal anesthesia for cesarean delivery between singleton and multiple gestation pregnancies [40]. Consistent with these observations, dose-finding data indicate that norepinephrine infusion requirements are similar in singleton and twin pregnancies [41^■■^]. In a randomized, double-blind trial, the estimated ED_50_ and ED_90_ values for prophylactic norepinephrine infusion were 0.022 (95% CI: 0.016–0.027) and 0.054 μg/kg/min (95% CI: 0.044–0.079) in singletons, and 0.023 (95% CI: 0.017–0.029) and 0.058 μg/kg/min (95% CI: 0.046–0.085) in twin pregnancies [41^■■^]. No significant differences were observed in prophylactic infusion rates, hemodynamic changes, maternal side effects, or neonatal outcomes [41^■■^].

Multiple gestation markedly increases the risk of uterine atony and PPH after cesarean delivery [42^■^]. Recent dose-finding studies indicate an oxytocin ED_90_ greater than 4 IU, supporting the administration of a minimum 5 IU bolus followed by maintenance infusion, rates higher than those typically required for singleton pregnancies [43^■■^]. Following the initial 5 IU bolus, early initiation of a maintenance infusion is recommended to maintain adequate uterine contractility and reduce the incidence of PPH in twin pregnancies [44]. Evidence from randomized and observational studies shows that long-acting uterotonics such as carbetocin may further reduce PPH risk although the optimal dosing strategy remains uncertain [45^■^,^46^,^47^]. Overall, current data suggest that uterotonic requirements are greater in multiple gestation pregnancies, but further trials are needed to refine the most effective prophylactic uterotonic strategy.

## CONCLUSION

Malpresentation and multiple gestation require vigilance and proactive analgesic and anesthetic management, both for vaginal delivery and cesarean delivery. There remain notable gaps in recent evidence and clinical practice, particularly regarding neuraxial analgesia use for ECV and the active management of labor epidural analgesia in malpresentation and multiple gestation. Recent studies highlight that neuraxial analgesia improves the comfort and success of ECV, neuraxial analgesia supports safer vaginal delivery of multiple gestation pregnancies, and cesarean delivery for multiple gestation carries an increased risk of uterine atony with higher oxytocin requirements. Further research is warranted to refine anesthetic techniques for these high-risk scenarios. Ultimately, the anesthesia team should anticipate rapid changes in the delivery plan, maintain readiness for urgent cesarean delivery, and tailor analgesic strategies to balance maternal comfort while optimizing maternal and neonatal outcomes.

## Acknowledgements

*None.*

## Financial support and sponsorship

*None.*

## Conflict of interest

*None.*
